# A Narrative Review Discussing Vasectomy-Related Impact upon the Status of Oxidative Stress and Inflammation Biomarkers and Semen Microbiota

**DOI:** 10.3390/jcm12072671

**Published:** 2023-04-03

**Authors:** Bogdan Doroftei, Ovidiu-Dumitru Ilie, Radu Maftei, Ioana-Sadyie Scripcariu, Theodora Armeanu, Irina-Liviana Stoian, Ciprian Ilea

**Affiliations:** 1 Faculty of Medicine, University of Medicine and Pharmacy “Grigore T. Popa”, University Street no 16, 700115 Iasi, Romania; 2 Clinical Hospital of Obstetrics and Gynecology “Cuza Voda”, Cuza Voda Street no 34, 700038 Iasi, Romania; 3 Origyn Fertility Center, Palace Street, no 3C, 700032 Iasi, Romania; 4 Department of Biology, Faculty of Biology, “Alexandru Ioan Cuza” University, Carol I Avenue no 20A, 700505 Iasi, Romania

**Keywords:** vasectomy, oxidative stress, inflammation, semen microbiota, humans, rats, mice

## Abstract

Background: Male contraceptive approaches besides tubal sterilization involve vasectomy and represent the method of choice among midlife men in developing countries thanks to many advantages. However, the subsidiary consequences of this intervention are insufficiently explored since the involved mechanisms may offer insight into a much more complex picture. Methods: Thus, in this manuscript, we aimed to reunite all available data by searching three separate academic database(s) (PubMed, Web of Knowledge, and Scopus) published in the past two decades by covering the interval 2000–2023 and using a predefined set of keywords and strings involving “oxidative stress” (OS), “inflammation”, and “semen microbiota” in combination with “humans”, “rats”, and “mice”. Results: By following all evidence that fits in the pre-, post-, and vasectomy reversal (VR) stages, we identified a total of n = 210 studies from which only n = 21 were finally included following two procedures of eligibility evaluation. Conclusions: The topic surrounding this intricate landscape has created debate since the current evidence is contradictory, limited, or does not exist. Starting from this consideration, we argue that further research is mandatory to decipher how a vasectomy might disturb homeostasis.

## 1. Introduction

Vasectomy is an elective and relatively minor family planning (FP) contraceptive approach that implies surgical ligation and disruption of the sperm flow from the proximal to the distal end of the vas deferens, similar to tubal sterilization, but less complicated than in women. From the health payer’s perspective, this is marked by low costs, effectiveness, toleration, and simplicity. Since no absolute contraindications are postulated, the exact figures fluctuate, with approximately one hundred million interventions performed worldwide based on the available statistics [1,2,3,4].

Though it has become typical and increasingly preferred among midlife men from growing and developing countries, and has a high success rate that reaches up to 99.7% and presumably a low risk of complications, complete reconstruction of the reproductive function remains under debate [5]. Around 10% of men, who represent a small fraction in contrast with the number of procedures, might display pain, bleeding, and inflammation, with vasovasostomy retaining a patency rate from 80 to 99.5%. Thus, it reflects the interest in VR correlated to other life-related variables such as divorce or remarriage [6,7].

Irrespective of the type, subsequent investigations discuss the impact on the testes, characterized by structural damage, because a surfeit of pathologies following vasectomy has been described with the expansion of our knowledge and understanding of this topic. Therefore, as evidence materializes to complete this spectrum and overall sphere of data, distinct mechanisms proposed to explain the causality started to gain interest and promoted a tremendous body of literature [5,6,8,9].

Precisely, these revolve around the increase of anti-sperm auto-antibodies production and hydrostatic pressure, but the most important is regarding OS, mainly because of fulminant generation of both reactive oxygen species (ROS) and reactive nitrogen species (RNS) [10]. OS is defined as an imbalance between anti- and pro-oxidants, the latter being found in elevated levels under the presence of ROS, which are unstable byproducts of normal metabolism, molecules via the acquaintance of electrons from proteins, lipids, nucleic acids becoming stable and able to initiate chain reactions that can harm the cells [11,12,13,14].

Under physiological situations when the level is low to moderate, ROS fulfills crucial biological functions by maintaing the cellular homeostasis, intracellular signaling pathways regulation, and immune and mitogen responses [15]. In this context, ROS ensure sperm capacitation, hyperactivation, and fertilization properties, motility, and chemotaxis; it promotes chromatic compaction in maturing spermatozoa and acrosome reaction and oocyte interaction [10,16,17,18].

With two documented sources of endogenous ROS, particularly leukocytes and immature spermatozoa [16,19], spermatozoa are susceptible to oxidation because of a lack of cytoplasmic antioxidant enzymes and a large amount of membrane unsaturated fatty acids [20]. Consequently, an impaired sperm function due to high ROS generation causes deoxyribonucleic acid (DNA) fragmentation, lipid peroxidation, membrane integrity loss, increased permeability, reduced motility, and apoptosis [11,12,13,14,16].

Fortunately, aerobic biological organisms are equipped with an antioxidant system present in seminal plasma and spermatozoa and of which intrinsic protection is provided by intracellular non- and enzymatic antioxidants [21]. The main contributors are catalase (CAT), superoxide dismutase (SOD), and glutathione peroxidase (GPx), while non-enzymatic examples are glutathione, carnitine, carotenoids, urate, and vitamins C and E, dedicated to maintaining redox balance and to avoid injury [22].

In contrast, an altered redox status of the seminal fluid has harmful effects on sperm parameters, which may culminate in male infertility [22,23,24,25,26] and thus could explain primary and secondary infertility, which accounts for approximately 25% of all idiopathic infertility cases besides those that range from 40 to 88% [27]. Conclusively, OS mediates cell death and tissue injury since it displays a pathological role originating from intrinsic etiologies such as inflammation triggered by exogenous factors and disorders [28,29,30,31].

Unfortunately, there is a lack of consensus on whether patients should be or not be examined for OS. The type of test used, and concerns over the antioxidant therapy duration and dose on this subject have sparked several controversies over the years [32,33]. This matter should be recognized as a public crisis mainly because it affects overall health [34,35,36], quality of life (QOL) [37,38,39], and life expectancy [40,41].

## 2. Methodology

The structure of the present manuscript follows the work of Green et al. [42] concerning writing a narrative review.

### 2.1. Rationale for Considering Semen Microbiota

“Microbiome” is a term introduced by Joshua Lederberg 22 years ago [43], subsequent molecular studies mapping four major ecosystems [44], including the urogenital microbiota. More specifically, semen microbiota refers to a diverse community of microorganisms that colonize and inhabit semen and include bacteria, viruses, fungi, and protozoa [45,46]. As each individual possesses their own unique personalized profile of microorganisms, a number of factors may shape and influence the microbiome, such as age, diet, lifestyle, and sexual behavior [47]. Recent studies have shown that semen is not a sterile fluid, as was previously believed, but rather contains a complex microbial ecosystem. Thus, recent data suggest that urogenital dysbacteriosis may be associated with male infertility and other reproductive health issues [48,49].

While many consider this topic a novel field of investigation that benefits from extensive attention since seminal OS, bacteriospermia, and leukocytospermia are known to play a major role in male infertility, a significant portion of cases remain idiopathic in nature [50]. Additional studies into this area are mandatory [51], as this approach has potential despite probiotics’ lack of efficiency [52]. To better emphasize the importance of the microenvironment that reunites communities at the level of the reproductive system stands the overexpression of the S-adenosyl-L-methionine (SAM) metabolite in infertile men. Genomic alterations in SAM lead to several observations, suggesting the role in various biological processes such as OS, DNA methylation, and polyamine synthesis could ensure the bridge to decipher this intricate interconnection [53]. Considering that semen microbiota in male reproductive health is still not well understood, additional research is needed to characterize the microbiota and comprehend its influence.

### 2.2. Databases Searches and the Strategies Applied

**Figure 1:**
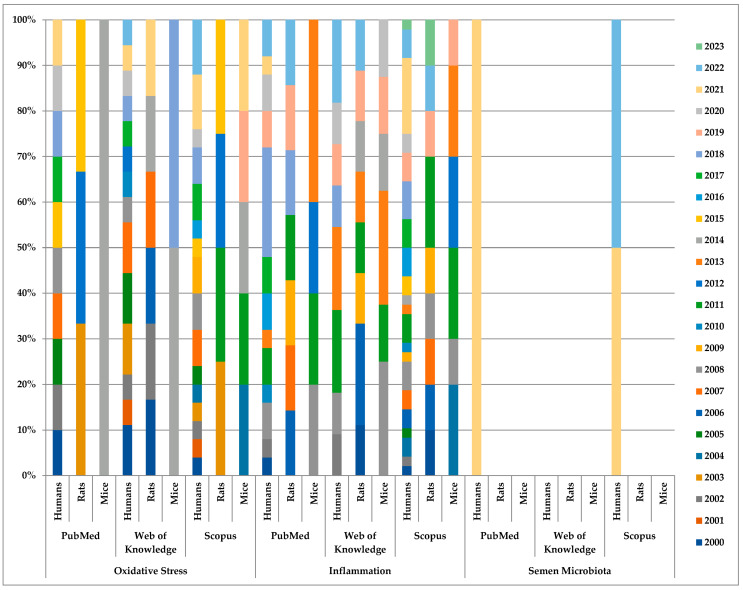
Diagram presenting the study percentage per year of publication based on the academic database, keywords, and model category.

All relevant information for conducting this manuscript was gathered this year between 1 January and 31 March from three academic databases, mainly PubMed, Web of Knowledge, and Scopus. We applied a controlled vocabulary to ensure optimum coverage of a significant body of literature that includes keywords targeting investigations carried out on “humans” and experimental models such as “mice” and “rats”. Irrespective of the organism, we coupled this string with “OS”, “inflammation”, and “semen microbiota”, having as a primary endpoint the associated consequences derived due to “vasectomy” with emphasis on histological alterations and biochemical and molecular changes (Figure 1).

### 2.3. Inclusion Criteria

Studies had to be research articles that report original data written in English, be published within the time frame 2000–2023, and describe the outcomes of all three stages of intervention (pre-, post-, and VR).

### 2.4. Exclusion Criteria

The following types of papers were excluded: (I) articles that report findings in another language than English and/or from another organism than those mentioned above, (II) case report, (III) case series, (IV) review, (V) systematic review, (VI) meta-analysis, (VII) letter to the editor, (VIII), editorial, (IX), correspondence, (X) opinion, (XI) response, (XII) comment, (XIII) work protocol, (XIV) trial, (XV) conference poster and/or abstract, (XVI) computational simulation, and (XVII) preprint.

### 2.5. Study Selection

Four independent authors (B.D., O.-D.I., T.A., and I.-L.S.) first screened the titles and abstracts of each initial study, and if considered eligible, we further proceeded with a full review of the content. Discrepancies or divergent opinions were solved by common consent between each team member who searched the studies and in parallel with the remaining three authors, R.M., I.-S.S., and C.I.

### 2.6. Limitations of the Study

Due to the nature of this manuscript and the desire to pursue a narrative review and the heterogeneity grade in the design and aim of studies, we were limited to a strictly objective and critical evaluation where possible to expose the main aspects surrounding this topic to offer an alternative, possibly comprehensive overview.

We identified at the initial evaluation n = 118 citations, from which n = 55 were excluded due to the following considerations: n = 32 reviews, n = 6 case reports, n = 4 articles were written in another language (n = 1 in Chinese, n = 1 in French, and n = 2 in German), n = 3 book chapters, n = 3 news, n = 2 letters, n = 2 correspondences, n = 1 editorial, n = 1 meta-analysis and n = 1 trial. From the remaining n = 63 studies, n = 32 were integrated into the “humans” studies category, whereas n = 31 were in the “mice/rats” arm.

**Figure 2:**
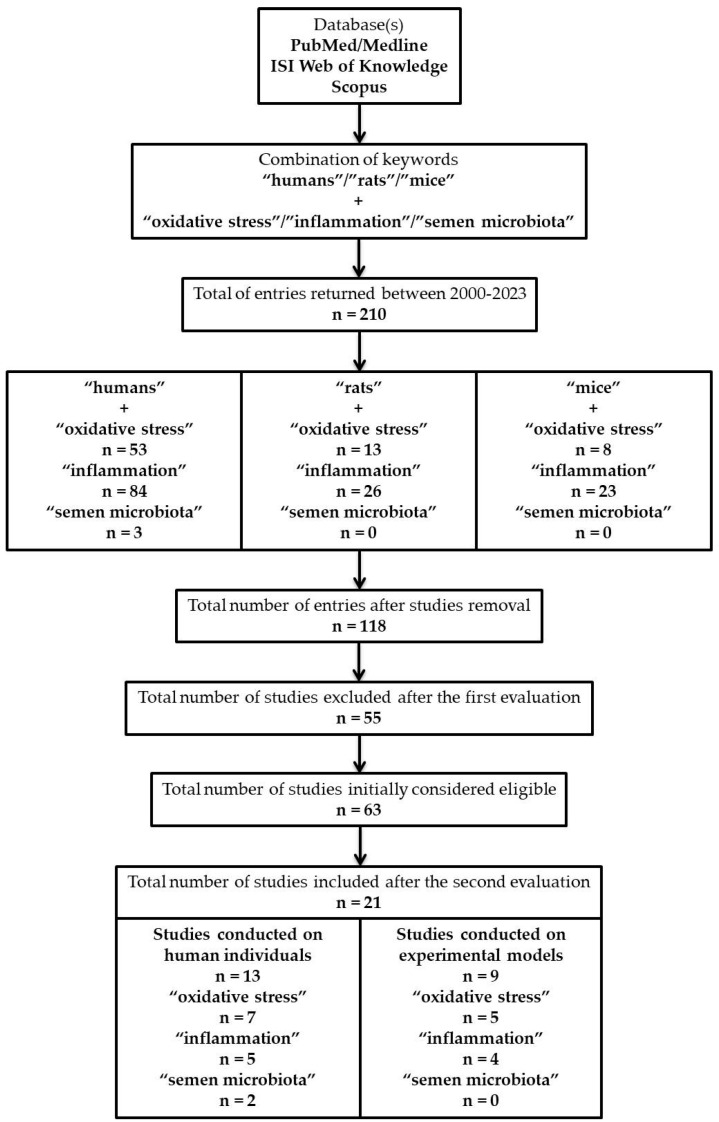
Flowchart diagram presenting the study design with the academic databases(s) searched, keywords applied, and strategy, number of entries, and studies eligible.

Sequentially, from n = 32 “humans” manuscripts, n = 19 were subsequently removed due to the degree of divergence, from which only n = 13 were included. Based on the study design, n = 7 targeted OS, n = 5 inflammation, and n = 2 the semen microbiota. It should be mentioned that n = 1 contains data valuable for 2 out of 3 main subsections of this manuscript. Regarding the experimental models, we identified only n = 9 eligible studies, and n = 22 were removed due to heterogeneity between the articles. Precisely, n = 5 were oriented toward OS, n = 4 toward inflammation, and n = 0 toward the participation of the semen microbiota (Figure 2).

## 3. Results

### 3.1. Oxidative Stress

#### 3.1.1. Humans

Zini et al. [54,55] conducted on two distinct occasions several investigations regarding the enzymatic activity of SOD and CAT in men that presented to undergo a vasectomy, post-vasectomy, or infertility evaluation. The authors demonstrate relatively similar values of SOD and CAT among the analyzed men. It can be argued that infertility might result from abnormal ROS generation rather than a defective activity of the antioxidant system. Although we cannot exclude the possibility of low semen antioxidants in subsets of patients with elevated ROS production, SOD and CAT appear to have a post-testicular origin, conferring protection to spermatozoa once in the female reproductive system. However, this raises the question of whether the antioxidant system of infertile men [54] is impaired in contrast with that of normospermic men [56,57] as an explicit diminishing in the presence of elevated ROS has not been observed. Thus, additional studies confirm a defective antioxidant system in infertile individuals [58,59]. The working protocols could play a prominent role in amplifying the risk of erroneous findings [60,61] drawn by the collection of seminal plasma and the presence of cellular debris [62], as a centrifugation speed at 400 g [63] to 700 g is insufficient.

Shortly after the observations made by Zini et al. [54,55] of a non-significant change in SOD and CAT, Pasqualotto et al. [64] extended the necessity of implementing strategies that target OS in infertile patients seeking specialty care. Not only ROS levels in the examined groups varied significantly; particularly in varicocele associated with infection, this further extrapolates to the total antioxidant capacity (TAC). ROS negatively influences sperm concentration, motility, and morphology irrespective of the clinical diagnosis, a presumably beneficial role involving the antioxidant supplements to reduce OS and improve sperm quality. The subsequent analyses suggest a notable difference in ROS, which mirrors negative correlations with sperm concentration, count, and motility in those who requested VR [64,65,66] and positive correlation with interleukin-6 (IL-6) [65] and leukocytes [66] in neat and washed semen. Cumulatively, all these changes should be confirmed in randomized controlled trials (RCTs) as possibly leading to subfertility.

A reference system development in accordance with the World Health Organization (WHO) directives by Athayde et al. [66] shows high ROS levels in non-leukocytospermic and leukocytes samples in washed and neat semen. Attempts to create a reference system led to different results because the data from Nallella et al. [67] were similar in values to those of Shekarriz et al. [68]. The optimal cutoff of 10.0 and 51.5 and the accuracy varied from 69 to 73.2% for samples with leukocytes and without leukocytospermia [66]. One major player through which can be deciphered the underlying OS action is age, considering the persistence of sperm parameters, as suggested by Cocuzza et al. [69] and by other teams [64,66]. Taken together, reference values of ROS in a fertile population can help pinpoint ROS pathologic interval. Moreover, it might pave the way for patients to benefit from antioxidant treatment in cases of delayed fatherhood or if couples with unknown causes of infertility desire to pursue assisted reproduction technology (ARTs). Although sperm concentration is a parameter that remains constant once with aging, irrespective of clinical diagnosis [64] and antioxidant enzyme activity [54], this irreversible process provokes a decrease in motility and morphology and warrants causality [64,69,70]. Varicocele associated with infection is a condition that amplifies up to fourfold ROS generation [64,71], which in part contradicts former results [69], generating elevation of lipid peroxidation [72,73] marked by inflammation and decrease of the fertilization rate [65,74].

Hypoxia in the testes activates the apoptosis process modulated by the transcriptional target of the p53 Bax gene upregulated in varicocele. In turn, this promotes the expression of hypoxia-inducible factor 1-α (HIF-1α) and of vascular endothelial growth factors (VEGF) in vascular endothelium and cytoplasm of germ cells, likely to have a paracrine effect on testicular microvasculature [75,76]. In parallel, VEGF can inhibit spermatogonial proliferation and instead enhance nitric oxide production, which perpetuate OS in varicocele individuals [77]. 4-hydroxynonenal (4-HNE) has come to attention because this aldehyde end byproduct of lipid peroxidation created during the oxidation of unsaturated fatty acids can provoke DNA damage and play a role in assembling adducts with proteins that induce apoptosis. Shiraishi et al. [78] highlight an association with the expression of p53 but that has an inverse effect with proliferating cell nuclear antigen (PCNA), spermatogonia, Sertoli cells, and primary spermatocytes. Though p53 mediates 4-HNE toxicity in varicocele and obstructive azoospermia (OA), this is only one pathway that deteriorates spermatogenesis, and the therapy may be helpful in those whom OS is responsible for subfertility [78]. Being hypothesized that OS could deteriorate in a gonadotropin-independent manner the proliferation of germ cells and implicitly spermatogonial proliferation [79] and spermatogenesis, research in the field reveals that 4-HNE-modified proteins did not act upon serum follicle stimulating hormone (FSH) nor Johnsen’s score but rather significantly on PCNA. That point sustains the idea that mitosis is susceptible to OS, as well as Sertoli cells or meiosis [78], which is in accord with another publication that did not show a relationship between serum FSH and PCNA expression [80]. Starting from that consideration, it was possible to bring into discussion the expression of p53 in association with germ cells apoptosis [81,82]. Staining protocols indicate that p53 is indeed involved in the apoptotic pathway because primary spermatocytes and spermatogonia both have been shown to be positive for p53, except bcl-2 associated X-protein (Bax) and terminal deoxynucleotidyl transferase dUTP nick end labeling (TUNEL) [82]. This further suggests a cell cycle arrest rather than apoptosis of p53 in spermatogonia.

A brief overview of the studies that comparatively discuss the effects upon OS biomarkers on human patients is detailed in Table 1.

#### 3.1.2. Experimental Models

Liu et al. [83] detail the role of the peroxiredoxins (Prxs) family in protecting spermatozoa [84], suppressing inflammation by oxidative damage induced by H_2_O_2_ and ultraviolet radiation (UV) [85], and alleviating germ cell apoptosis and histological alterations [83]. The OS-Prx 1, 2, 3, and 6 linkage could represent the foundation for antioxidant supplements administration to reduce damage inflicted by the vasectomy to the testes and epididymis and improve the fertility rate post-VR. As an essential physiological process, apoptosis maintains testicular homeostasis while the opposing lead to germ cell death [86]. The apoptotic cells are predominantly spermatogonia and elongating spermatids [83] in rats [87] and hamsters [88]. With degeneration of the seminiferous tubule that progresses in a time-dependent manner post-vasectomy, it may advance to more severe cases on days 15–30. A documented reduction in the number of spermatids and spermatocytes in mice [83,89] in week 5 [89] and recovery at 45–120 days [83] in humans last for one to two years [90], and up to three months in rats [91].

Thiobarbituric acid reactive substances (TBARS) or forms of RNS can hamper spermatogenesis in OA per Başar et al. [92]. However, malondialdehyde (MDA) is the primary biomarker of lipid peroxidation, which bears a role in sperm fluidity, serum estradiol (E2), and progesterone (P4). The level of MDA gradually increases throughout the next couple of weeks post-vasectomy [83] and persists for half a year after tubal ligation (TL) [93].

The MDA and prooxidant-antioxidant balance (PAB) levels appear to be dependent on the point of ligation, contrary to the proximal end of the vas deferens sectioning [94], due to intra-organ hydrostatic pressure following site obstruction nearest to the ductal obstruction. Not surprisingly, the diameter testes of vasectomized rats were notably smaller, mainly when the incision was performed closest to the testis but not the contralateral testis [94,95], a description of a severe contra-lateral injury being made [96]. Mechanically speaking, ROS fulminant generation might be connected to TL-induced hypoxia in the fallopian tubes (FTs) and ovaries by slowing down the electron transmission in the mitochondria [97]. It elevates the expression of inducible nitric oxide (iNOS) and VEGF [98] since hypoxia-inducible factors (HIF-α) bind to hypoxia-sensitive regions and initiate a response either to regulate VEGF transcription or mediate HIF-α by iNOS [98].

One mg/kg melatonin intra-peritoneally (I.P.) reduces testicular damage after twelve weeks of the administration as the testicular diameter and mean seminiferous tubular diameters (MSTD) of the contralateral testis remain unaffected [94]. The antioxidant treatment could be a recommended approach prior to sperm extraction for intracytoplasmic sperm injection (ICSI) due to obstructive infertility post-VR. Analogous correlations with humans for oxidative biomarkers, sperm motility, and morphology were discovered. In cases of negative associations, but instead between TAC with sperm morphology, proposed mechanisms toward infertility pinpointed oxygen radicals generation by leukocytes, sperm damage due to epididymal blockage, and of the immune system or immature sperm [73,99].

Nevertheless, there is a lack of evidence concerning seminal plasma ROS and TAC levels’ disparity between infertile and healthy men [73], as some studies suggest between VR and donors. Some reports indicate an elevated ROS in those that underwent unilateral vasectomy, showing histological changes in ipsilateral testicular tissue [100]. This is based on the observations that there is an abundant collagen fibril accumulation and anti-vimentin antibodies in the peritubular area and within myoid cells, accompanied by intense immunoglobulin G (IgG) antibodies in both testes [101]. Despite TBRAS increased level, no gross morphological change was remarked even though in the literature the increase of superoxide radical in the granuloma side post-vasectomy was noted [102].

Gale et al. [103] demonstrate that females seek refuge when near castrated counterparts compared with healthy or vasectomized mice. They exhibit increased levels of glucocorticoids, and this avoidance behavior suggests a response to stress rather than mating behavior.

A brief overview of the studies that comparatively discuss the effects upon OS biomarkers on rats and mice is detailed in Table 2.

### 3.2. Inflammation

#### 3.2.1. Humans

The physiological implication of resistin led to debate among the community. A publication from Kratzsch et al. [112] shows that adipose tissue-specific secretory factor (ADSF) positively correlates with inflammatory mediators IL-6 and elastase [113,114]. This cysteine-rich secretory protein upregulates the expression of IL-6 and leukocytes [115], with defined roles in inflammation and metabolism regardless of the semen quality [112]. The theoretical source resides within the mononuclear leukocytes [113] and Leydig cells [116]. Subsidiary data indicate a negative association with the body mass index (BMI) in non-obese patients with normal BMI [112,117] at a corresponding concentration [118], not in the pathological range for both IL-6 [119] and elastase [120].

Systemic inflammation transmission in obese patients with metabolic syndrome (MetS) recently brought forward the possibility of elucidating the impact on semen and the genital tract. Though Pilatz et al. [121] noted an increase of C-reactive protein (CRP), IL-6, and interleukin-10 (IL-10) by coverage of insulin-dependent tissue [122], this attempt to explain the causality remains provocative. With no change in semen parameters nor systemic inflammation based on IL-6 and IL-10 [121], data confirm numerous studies on the increase of circulating biomarkers [123,124,125,126,127,128,129] and have to do with ethnicity [130], gender [124], sample size [125,131], as well as definition and heterogeneity [128,131].

With a seminal cytokine concentration in the range [121] with a previous report [132] on polymorphonuclear (PMN) elastase and peroxidase-positive leukocytes [133,134], the ratio in blood and semen was comparable with the spectra of those with human immunodeficiency virus (HIV) [135]. The observed inflammation did not impact standard pointers of seminal tract inflammation. As BMI is not equivalent to MetS [134,136,137], the semen parameters and concentrations are constant, instead causing a decrease [134], and impact motility [134,138] and morphology [137] by amplifying the risk for oligozoospermia or azoospermia [139,140] in morbidly obese men.

Chemokine (C-C motif) ligand 20 (CCL20), chemokine (C-C motif) ligand 28 (CCL28), the glutamic acid-leucine-arginine (ELR) motif-negative MIG-chemokine (C-X-C motif) ligand 9 (CXCL9) [141,142,143] and more recently granulocyte chemotactic protein 2 (GCP-2)/chemokine (C-X-C motif) ligand 6 (CXCL6) [144] suggest that inflammation of the pharyngeal epithelial cells possess antimicrobial activity that is regulated by MIG/CXCL9 [145], both being synthesized in the male reproductive tract alongside ELR-negative/positive CXC chemokines [146]. Collin et al. [147] report antithetical levels of GCP-2/CXCL6 between healthy donors and vasectomized men in seminal plasma, which may offer support for those who desire elective sterilization. Conclusively, GCP-2/CXCL6 confers protection against the urogenital pathogen *Neisseria gonorrhoeae* or commensal gut bacteria *Enterococcus faecalis* by activating nuclear factor kappa-light-chain-enhancer of activated B cells (NF-κB) and downstream inflammatory stimuli as lipopolysaccharides (LPS), tumor necrosis factor-α (TNF-α), and interleukin-1β (IL-1β) [148]. There are organs whose epithelial cells’ GCP-2/CXCL6 are expressed during inflammation such as gastrointestinal epithelial, airway, or pharyngeal cells [148,149,150,151,152,153].

Belardin et al. [154] spotlighted a couple of months ago the limited knowledge regarding the purinergic signaling in the ductus efferentes. Damage-associated molecular patterns (DAMPs), including adenosine 5′-triphosphate (ATP) and uracil-diphosphate glucose (UDP)-glucose that initiates P2T14 localized in the apical membrane of epididymal epithelial cells, is triggered in inflammatory conditions if the germ cells are damaged. Independently of the pro-inflammatory receptor P2Y14′ expression in the proximal regions of ciliated cells and distal parts of unidentified epithelial cells, P2Y14 expression was further reported in a subpopulation of clear cells (CC), basal cells (BC), and also in principal cells from caput, epididymal, and corpus, and cauda regions. The mRNA was noted in the corpus, cauda, and principal cells (PC) of the corpus region in vasectomized men. This led to the conclusion that vasectomy causes spermatozoa congestion characterized by an inflamed-prone localized environment.

A brief overview of the studies that comparatively discuss the effects upon inflammatory biomarkers on human patients is detailed in Table 3.

#### 3.2.2. Experimental Models

Mice immunization with viable syngeneic testicular germ cells (TGC) led to epididymitis without orchitis development, contrary to targeted experimental autoimmune orchitis (EAO). Similar to in vivo studies that involved retrograde *Escherichia coli*-induced epididymitis, both Qu and Turner et al. [155,156] observe in the initial segment of the epididymis lymphocytic infiltration due to obstruction of vas deferens. Responsible for this inflammatory landscape [157,158] stands the myoid and epithelial cells of the epididymis associated with the exhibition of IL-6 and IL-10 in cryptorchid crypt epididymis [159]. While IL-6 expression in VR [65] increases, in parallel with IL-10 suppress EAO development [160], it can reduce TGC-induced EAO, and triggers apoptosis of germ cells within seminiferous tubules [161], known to exert opposite effects in autoimmune diseases [162,163,164,165].

Regulatory T (Treg) cells are viewed as powerful immunosuppressive canonical tolerance-inducing entities in peripheral organs, postmeiotic and meiotic cell antigens (MGCA) gaining tolerance by the development of antigen-specific T cells, double negative T cells (T^DNs^). They prevent autoimmune responses that are vasectomy-related in inflamed epididymis [166,167].

Apart from regular reported inflammatory stimuli, galectin-3 is a recent protein recognized as a macrophages-secreted inflammatory factor in vasectomized mice, whose expression increases after vasectomy in 40% of testicular tissues, particularly seminiferous tubules, interstitial tissues, and tunica albuginea. Besides the inflammation, vasectomy illustrate several degenerative alterations in the number of germ and Sertoli cells in contrast with sham-operated subjects, leading to the conclusion that vasecotmy induce chronic complications and infertility post-vasovasostomy per Kashani et al. [168].

a2NTD is a pivotal portion of the a2V protein at the N-terminal released from activated monocyte and secrete cytokines [169]. According to Jaiswal et al. [170], the protein ATPase H+ transporting V0 subunit A2 called Atp6v0a2 plays a crucial role in pregnancy which is not detected in caudal epididymis of non-capacitated spermatozoa but of capacitated murine and highly expressed in the acrosomal region. Throughout the peri-implantation, decidual macrophages that possess a high degree of plasticity vary depending on the gestation age and ensure changes in phenotype(s) replying to inflammatory responses, including leukemia inhibitory factor (LIF) and interleukins (IL-1β), and display polarization which is skewed toward M1 traits. However, the shifting into a mixed M1/M2 population types persist until mid-pregnancy after placental development to prevent rejection and impact parturition is promoted when the extravillous trophoblasts invade and attach to the uterine stroma and settle in the endometrial lining. In short, vasectomy may prevent further histological alterations and inflammation in experimental epididymitis. However, inflammation is a response initiated either by the autoreactive lymphocytes when gaining the access to TGC autoantigens against autoantigens of mature spermatids or localized in the preimplantation uterus by the influx of macrophages during the onset of pregnancy that may culminate in a successful pregnancy outcome.

The Hedgehog signaling pathway is essential in the epithelial-mesenchymal (Müllerian duct) communication, oviductal coiling, and uterine gland formation that interplays with Wnt 4 and Wnt 5 and homeobox A13 (HOXA13) signaling. In the female reproductive tract’s FT, no noticeable signs of masculinization and regular regression of the mesonephric duct in *Amhr2^cre/+^SmoM2* female mice were seen [171]. RNASE9 that resides within the cells in the proximal caput is not expressed in the cauda but rather in the sperm from the caput and corpus of males [172]. Around sixty-nine seminal fluid proteins (sfps) were identified after mating [173], macrophage population in the corpus luteum (CL) fulfilling an essential part in inflammatory and immune shield throughout sperm migration in the female tract [174]. The eventual depletion of host macrophages hampers *Chlamydia muridarum* dissemination. Even though it is a non-motile bacterium, it still invades testicular cell populations targeting Sertoli/Leydig and spermatogenic cells through the penile urethra [175]. As expected, the remarks made in the case of human patients apply to rodents as well. Seminal obstruction, regardless of the testes and side, reduces the rate of spermatogenesis, concluding that unilateral vasectomy induces vast histological changes that cover unilateral obstruction, intratubular germ cell necrosis, and an increase in tubular pressure probably linked to autoimmune responses [176].

A brief overview of the studies that comparatively discuss the effects upon inflammatory biomarkers on rats and mice is detailed in Table 4.

### 3.3. Seminal Microbiota

#### Humans

Irrespective of age, eating habits may disturb the microbial niches, particularly seminal microbiota. The so-called dysbiosis is a deleterious phenomenon that inflicts changes in the proportion of microbial communities diminishing core taxa. Owing to the fact that the underlying role of semen microbiota in infertility is obscure, Lundy et al. [53] conducted a pilot study to identify distinct bacterial signatures between non- and vasectomized men. The sequencing of the 16S ribosomal RNA (rRNA) of specific biological samples revealed a 2.3% similarity between the rectal swabs, semen, and urine samples. A 10% in terms of taxa ratio between the urine and semen among healthy individuals was also identified. In addition to the increase in α-diversity regarded as the diversity of species within a single sample or environment, the β-diversity refers to the differences in microbial community composition between multiple samples or microenvironments indicate disproportionality and marks a decrease of *Collinsella* and *Staphylococcus* after vasectomy. The rectum samples were populated by *Lachnospiraceae*, *Collinsella*, and *Coprococcus*, coupled with a reduction of *Anaerococcus* in infertile men. Conversely, *Anaerococcus* in the urine was increased, with a decrease of *Collinsella* and *Aerococcus* increment in semen of infertile men. Their work and results are consistent with previous studies that suggest a rich microbiome in both semen [177,178,179,180] and voided urine [181,182] with a global diversity shaped in a sex-dependent manner [177,183] and personalized profiles between the sites similar to what has been already sought in females [184]. Although shown to be depleted following a vasectomy, phylum *Firmicutes*, and *Actinobacteria* were identified in the testes of azoospermic men [185]. In this way, this completes the sphere concerning the inverse association between semen quality and *Prevotella* [180,186] and the presence of *Aerococcus* as a uropathogen in the semen of infertile men [53,177].

In a second pilot study conducted by Arbelaez et al. [187], they brought strong evidence of the decreasing α-diversity caused by vasectomy. There were identified changes in the *Paracoccus*, *Sphingomonas*, and *Brevundimonas* as they decreased following a vasectomy, while the abundance of *Corynebacterium* increased. This study reveals an increase in *Proteobacteria*, *Actinobacteria*, *Bacteroidetes*, and *Firmicutes* [185,187,188] and a decrease in the α-diversity with a particular accent on the operational taxonomic units (OTUs) and Hill1 diversity, which might be explained by the lack of testicular/epididymal microbial niche [189]. Nonetheless, an increase in *Actinobacteria* is documented in those with idiopathic nonobstructive azoospermia (NOA) [185,187], while semen samples tested both negative in three out of five and positive in five out of five before and after vasectomy [190].

As a concluding remark, both these authors discuss the necessity of additional and comprehensive investigations as conflicting results in terms of α-diversity are given. Until that point, the limitations of these studies are the relatively small sample size and ethnic diversity for which additional longitudinal studies could elucidate the causality. A brief overview of all changes that were noted among microbial ratio at the level of semen can be found in Table 5.

## 4. Discussion

Infertility evolved into a profound issue in many countries affecting a significant proportion of individuals of both sexes as the popularity of ART procedures matures and tries to meet the requests. As far as it goes for men, many non(reproductive) disorders and treatments might stand behind this, while a fraction of cases remain unexplained and, thus, a thorough evaluation could guide the clinicians to treatable causes and allow natural fertility [191,192].

The popularity of microsurgical reconstruction has increased over the years among men who undergo a vasectomy as it is more cost-effective compared with in vitro fertilization (IVF) and ICSI or sperm retrieval [193]. However, conditions in such cases are that vasectomy was performed less than fifteen years prior and there are no female risk factors, as female age and obstruction epididymal are of interest, and the procedures should be individualized [194].

Varying sperm retrieval procedures are well-established to obtain spermatozoa for ICSI in infertility settings. Despite the tendency to treat azoospermic patients by ICSI using surgically retrieved sperm, vasovasostomy remains the gold standard. The current technology ensures spermatozoa recovery in approximately half of the patients with NOA. Otherwise they can be relatively easily obtained by testicular sperm aspiration (TESA), percutaneous epididymal sperm aspiration (PESA), or testicular biopsy in men in OA when vasovasostomy is not recommended [195,196]. For about half of NOA in which the sperm may be obtained by testicular biopsy, there are no accurate tests to predict the recovery rate, which is otherwise limited and the chance to establish an ongoing pregnancy as well is decreased relative to those with normal spermatogenesis [197].

Reports indicate that repeated PESA punctures (up to five) with a 25G needle induce a cumulative effect dependent on the number of sessions. This is characterized by inflammatory and stereological alterations that reunite under local fibrosis, lymphoplasmocitary infiltrate, and volumetric enlargement of the connective tissue [198]. Post-vasectomy pain syndrome (PVPS) that reunites conditions mentioned above may be exceeded by using anti-adhesion agents that confer protection against further structural destruction post-operatively [199]. Recent advances in the field that rely on biodegradable polymer grafts offer insight and offer an effective response in reconstructing obstructed or absent vas deferens [200]. Additional screenings in the aftermath of vasectomy pinpoint at the ligation site acute inflammation and augment the expression of transferrin and osteopontin by the vas and epididymal epithelium [201]. In cases of drug-induced epididymal inflammation, this may pave the path of measuring the induction of pro-inflammatory genes [202], vasectomy leading to sperm antibodies, and sperm granuloma at least [203].

Testicular sperm extraction (TESE) for NOA and OA are also widely conducted with OA patients representing the group of good candidates for seminal reconstruction or microscopic epididymal sperm aspiration (MEA) [204]. Contrary to expectations following TESE, this indicated it would be necessary to turn to a sperm donor or adoption. As suggested, microepididymal sperm aspiration (MESA) enhances the chances of pregnancy and clinical deliveries after ICSI without testicular surgical damage [205].

ICSI using round spermatids is not a suitable option, with a means to improve the outcome consisting in in vitro cultures of immature germ cells until more advanced stages. Consequently, the low pregnancy rates reflects the need for much more safer approaches [206,207,208] and we can witness a decline in the percentages of pregnancy rates via ART. The number of spermatids, testicular abnormalities, and apoptosis of germ cells post-vasectomy may worsen with extended periods of obstruction [209] and is viewed in the pregnancy rates.

Knowing that spermatogenesis usually occurs in larger testis, a unilateral therapeutic biopsy in OA patients may be pursued if the size difference between the testes is significant [210]. A randomized clinical trial published several years ago aims to improve the outcomes following VR by using intra-operative local mitomycin-C (MMC) to prevent common sexual complications, late stricture, and obstruction. Among several advantages that refer to costs and increase in the sperm count, intra-operative MMC appears to be safe and efficient in vasovasostomy in the first five to ten years [211].

It is worth mentioning that clinical trials carried out over the years ruled out the possible increased risk of corpora amylacea (CA) [212] and atherosclerotic heart disease, despite there being remarked a significant proportion of antisperm antibodies (ASA) development after vasectomy [213]. No correlation exists between ASA and inflammatory/infectious diseases [214] in defiance of the positive detection rate of IgG and immunoglobulin A (IgA) [215].

Varicocele is a dilation of the pampiniform plexus of the spermatic cord and the most common and correctable cause of male infertility, defined by a decline in sperm production and quality [216,217]. Varicoceles presence is a pointer of high predisposition to diabetes, hyperlipidemia, and cardiovascular conditions than in those who already have undergone vasectomy [218]. From the reduction in the spermatids/Sertoli cell ratio per unit area without influencing the number of Sertoli cells [209], varicoceles negatively influence sperm parameters secondary to elevated levels of OS, fragmentation of mitochondrial, and of genomic DNA, and apoptosis to necrosis and smaller testes of the varicoceles localization side [219,220].

Multiple meta-analyses on sperm DNA fragmentation clearly show significantly elevated DNA damage [221], lower pregnancy rates, abnormal embryo evolution, and risk of recurrent pregnancy loss (RPL) as the significance of sperm DNA fragmentation (SDF) is still debatable [222,223,224]. Fortunately, surgical repair is associated with an improvement in sperm DNA and those who underwent this procedure have benefited from a low sperm DNA fragmentation [225]. DNA fragmentation is significantly higher in VR individuals without impacting spontaneous or ART pregnancy rates since there are negative correlations between DNA fragmentation index (DFI) and spermiogram [226]. Creatine kinase (CK) could be considered as an indicator of sperm quality and maturity as its level was up to 18-fold higher in the severely oligospermic group than in mild/moderate and compared with donors [227].

Recent studies demonstrated that spermiogenesis is the final stage of spermatogenesis in infertile patients with varicocele, and they are more likely to retain cytoplasmic droplets (CD). This morphologic feature related to high levels of ROS generated inside the seminiferous tubules by the CD in immature spermatozoa is involved in DNA damage [228,229]. A hypothetical mechanism originates from the overexpression of anaerobes in varicocele since both OS and leukocytospermia slightly correlate in the semen of infertile men [53,230].

## 5. Conclusions

Vasectomy, as we extensively discussed throughout this manuscript, can induce OS that manifests under a specific grade of inflammation and can perturb the host’s eubiosis. Although direct correlations are yet to be discovered, we tried to emphasize how impactful and deleterious such an intervention can be and in which manner it can perturb homeostasis. Unfortunately, the wider consequences of vasectomy upon an individual’s health have not been a subject intensively debated. Instead are better marked by experiments carried out in the past two decades because of numerous of the contradictions that arose and the fact that such analyses are not included in the clinical routine. In conclusion, our primary goal was to comprehensively ensure by paving the road for future studies and would be helpful to other teams conducting investigations in this context.

## Figures and Tables

**Table 1:** Stratification of studies per year of publication based on design and allocation of patients with the main results that highlight changes in OS biomarkers in human individuals.

Total Number of Patients and Allocation	TAC (Trolox Equivalents)	ROS Log (ROS + 1) (cpm)	SOD (U/mL)	CAT (U/mL)	*p*-Value	Method of Assessment	Year of Publication and Reference
n = 117 n = 12 fertile men n = 21 azoospermic men n = 84 nonazoospermic men	-	-	37.0 ± 2.8 v. 37.1 ± 2.4 v. 49.1 ± 1.6 ^a^	369 ± 49 v. 364 ± 30 v. 316 ± 1.9	SOD 0.03 ^b^ CAT 0.35 ^c^	SOD—inhibition of the NBT reduction; absorbance at 545 nm and measurements at 3 to 5 min [57] CAT—decrease in the concentration of H_2_O_2_; absorbance at 630 nm and measurements 10 min after alkalinization [57]	2000 [54]
n = 186 n = 19 controls n = 77 varicocele n = 43 vasectomy reversal n = 36 idiopathic infertility n = 11 varicocele associated with infection	1653.98 ± 115.29 v. 1173.05 ± 58.07 v. 1532.02 ± 74.24 v. 1014.75 ± 79.22 v. 1026.33 ± 150.32	1.3 ± 0.3 v. 2.2 ± 0.13 v. 2.4 ± 0.17 v. 2.3 ± 0.21 v. 3.2 ± 0.25	-	-	TAC - 0.0005 0.40 0.0001 0.002 ROS - 0.006 0.001 0.006 0.0001	ROS—chemiluminescence measurement with a luminometer; measurements for 15 min TAC—enhanced chemiluminescence assay; measurements for 100 s [73]	2000 [64]
n = 27 n = 16 fertile men n = 11 post-vasectomy men	-	-	37 ± 10 v. 36 ± 10	389 ± 163 v. 325 ± 119	SOD 0.69 b CAT 0.22 ^c^	SOD—inhibition of the NBT reduction; absorbance at 545 nm and measurements at 3 to 5 min [57] CAT—decrease in the concentration of H_2_O_2_; absorbance at 630 nm and measurements 10 min after alkalinization [57]	2002 [55]
n = 37 n = 15 healthy donors n = 22 vasectomy reversal	1,556.4 ± 468.1 v. 1719.8 ± 758.6	1.2 ± 0.7 v. 2.3 ± 0.97	-	-	TAC 0.69 ROS 0.009	ROS—chemiluminescence measurement with a luminometer; measurements for 15 min TAC—enhanced chemiluminescence assay; measurements for 100 s [71]	2005 [65]
n = 160 n = 114 fertile men n = 46 subfertile men	-	ROS in neat semen ^d^ 0.4 v. 1.5 ROS in washed semen ^d^ 8.1 v. 116	-	-	ROS in neat semen <0.0001 ^e^ ROS in washed semen <0.0001 ^e^	ROS in washed and neat semen—chemiluminescence with a luminometer; measurements for 15 min	2007 [66]
n = 47 patients n = 20 varicocele n = 10 OA n = 17 idiopathic oligozoospermia or azoospermia	4-HNE				p53—varicocele <0.01 p53—OA <0.05	-	2007 [78]
n = 144 n = 46 controls n = 20 ≤ 40 years old n = 78 < 40 years old	-	ROS in neat semen ^d^ 0.68 v. 0.29 v. 1.51	-	-	A 0.0009 ^f^ B 0.006 ^f^ C < 0.0001 ^f^	ROS in neat semen—chemiluminescence with a luminometer; measurements for 15 min	2008 [69]

NBT—nitroblue tetrazolium, a—*p* < 0.05; Student–Newman–Keuls one-way analysis of variance (ANOVA), b—*t*-test, c—Mann–Whitney rank-sum test, d—20 x 10^4^ cpm, e—*p* < 0.05; Mann–Whitney *U*-test, A—*p*-value between ≥ 40-year-old fertile men and <40-year-old fertile men, B—*p*-value between ≥ 40-year-old fertile men and controls, C—*p*-value between < 40-year-old fertile men and controls, f—Wilcoxon rank sum test.

**Table 2:** Stratification of studies per year of publication based on design, allocation, and strain with the main results that highlight changes in oxidative stress biomarkers in rats and mice.

*Rats*					
Number of Experimental Models and Allocation	Strain	Oxidative Biomarker(s)	*p*-Value	Method of Assessment	Year of Publication and Reference
n = 20 n = 10 vasectomy group n = 10 sham group	Sprague-Dawley	nitrate/nitrite (µmol/g protein) 35.6 ± 3.1 v. 19.3 ± 0.7 TBARS (nmol/g protein) 3.7 ± 0.1 v. 3.1 ± 0.1	nitrate/nitrite 0.001 TBARS 0.001	nitrate/nitrite—absorbance at 540 nm [104] TBRAS—fluorescence—excitation at 510 nm and emission at 553 nm [105] with modifications from [106,107]	2006 [92]
n = 80 n = 10 controls per group n = 10 intact rats n = 10 ligation between *caput epididymis* and upper pole of the testis n = 10 ligation to both ends (proximal and distal) after division of vas deferens 2–3 cm from *caudal epididymis* n = 10 vas divided 2–3 cm from *caudal epididymis* and ligation applied only to the proximal end	Sprague-Dawley	MDA (µmol/mg protein) group I 0.38 ± 0.24 v. 0.38 ± 0.07 group II 0.92 ± 0.16 v. 0.47 ± 0.23 group III 0.69 ± 0.02 v. 0.42 ± 0.14 group IV 0.40 ± 0.21 v. 0.38 ± 0.02	<0.05	MDA—modified TBA method of [108]; absorbance at 535 nm	2011 [94]
n = 56 n = 28 sham group (sham-vasectomy/sham-TL) n = 28 vasectomy group (vasectomy/TL)	Sprague-Dawley	PAB (HK unit) MDA (mmol/mL)	<0.05	PAB—enzymatic reaction—chromogen TMB oxidation chemical reaction—TMB cation reduction MDA—fluorescence [109]; absorbance at 530 nm	2012 [93]
	***Mice***				
n = 112 n = 56 vasectomy group n = 56 sham group	Kunming	MDA (nmol/mg protein)	day 15 <0.05 day 30 <0.01	-	2014 [83]
n = 70 n = 20 females exposed to vasectomized males n = 20 females sham group n = 20 females full castration n = 10 females C57BL/6	*Mus musculus* C57BL/6	liver, kidney, heart, and gastroc thiols - liver, kidney, heart, and gastroc aconitase	housing partner thiols liver 0.895 kidney 0.248 heart 0.524 gastroc 0.724 aconitase liver 0.487 kidney 0.288 heart 0.729 gastroc 0.469 refuge present/ absent thiols liver 0.616 kidney 0.112 heart 0.745 gastroc 0.334 aconitase liver 0.320 kidney 0.967 heart 0.636 gastroc 0.245	acid-soluble thiols [110]; modified colorimetric method from [111]	2019 [103]

TBA—thiobarbituric acid, TMB—tetramethylbenzidine 3, 3′, 5.5′.

**Table 3:** Stratification of studies per year of publication based on design and allocation of patients with the main results that highlight inflammation in human individuals.

Total Number of Patients and Allocation	Inflammatory Biomarker	*p*-Value	Method of Assessment	Year of Publication and Reference
n = 37 n = 15 healthy donors n = 22 vasectomy reversal	IL-6 (pmol/mL) 109.7 v. 4.4 Log_10_ (IL-6 + 1) 0.99 ± 0.97 v. 2.1 ± 0.9	IL-6 (pmol/mL) - Log_10_ (IL-6 + 1) 0.007	double-antibody sandwich ELISA; measurement at 410 nm [74]	2005 [65]
n = 54 n = 21 normozoospermic men n = 33 pathozoospermic	IL-6 (pg/mL) 11.9 v. 12.0 Total IL-6 (pg/ejaculate) 47.6 v. 46.4	resistin—elastase ≤0.00001 resistin—IL-6 <0.01	ELISA resistin—sensitivity—0.9 ng/mL; intra and inter-assay CV below 8.9% elastase and IL-6—sensitivity—2.0 ng/mL; intra and inter-assay CV below 6.9%	2008 [112]
n = 21 n = 14 fertile men n = 7 vasectomized men	GCP-2/CXCL6	0.03	ELISA intra-assay precision 5% inter-assay reproducibility 7%	2008 [147]
n = 54 n = 27 controls n = 27 MetS	IL-1α 5.53 v. 5.01 IL-1β 1.36 v. 2.40 IL-6 14.93 v. 13.95 IL-12p70 0.95 v. 0.00 TNF-α 1.65 v. 1.23 IFN-γ 3.13 v. 0.00 IL-2 0.51 v. 0.00 IL-4 0.05 v. 0.00 IL-5 3.83 v. 7.54 IL-7 463.98 v. 378.76 IL-9 1.68 v. 0.00 IL-10 0.92 v. 0.96 IL-13 0.025 v. 0.00 IL-17A 0.19 v. 0.00 VEGF 325.16 v. 302.33 FGF 4.38 v. 0.00 GCSF 5.16 v. 2.69 GMCSF 1.34 v. 0.66 Granzyme A 6.92 v. 8.343 MCP-1 8582.33 v. 5090.51 MIP-1α 4.42 v. 1.90 MIP-1β 77.78 v. 44.40 RANTES 125.68 v. 99.03 Eotaxin 41.81 v. 30.17 IL-8 2300.30 v. 1538.18 MIG 10503.09 vs 4407.00 IP-10 10201.11 vs 2160.75 I-TAC 4390.74 vs 4608.28 FKN 154.80 vs 170.80	IL-1α 0.872 IL-1β 0.786 IL-6 0.680 IL-12p70 0.763 TNF-α 0.338 IFN-γ 0.420 IL-2 0.577 IL-4 0.404 IL-5 0.580 IL-7 0.137 IL-9 0.632 IL-10 0.691 IL-13 0.403 IL-17A 0.150 VEGF 0.904 FGF 0.349 GCSF 0.374 GMCSF 0.303 Granzyme A 0.830 MCP-1 0.243 MIP-1α 0.396 MIP-1β 0.159 RANTES 0.387 Eotaxin 0.185 IL-8 0.345 MIG 0.016 ^a^ IP-10 0.012 ^a^ I-TAC 0.951 FKN 0.736	CBA LLOD 1.0 2.3 1.6 0.6 0.7 1.8 11.2 1.4 1.1 0.5 3.1 0.13 0.6 0.3 4.5 3.4 1.6 0.2 3.7 1.3 0.2 0.8 0.002 0.8 1.2 1.1 0.5 9.1 22.3	2017 [121]
*				2022 [154]

ELISA—enzyme-linked immunosorbent assay, CV—coefficients of variation, CBA—cytometric bead array, LLOD—lower limit of detection, IL-1α—interleukin-1α, IL-1β—interleukin-1β, IL-12p70—interleukin-12p70, IFN-γ—interferon-γ, IL-2—interleukin-2, IL-4—interleukin-4, IL-5—interleukin-5, IL-7—interleukin-7, IL-9—interleukin-9, IL-13—interleukin-13, IL-17A—interleukin-17A, FGF—fibroblast growth factor, GCSF—granulocyte colony-stimulating factor, GMCSF—granulocyte-macrophage colony-stimulating factor, MCP-1—monocyte chemoattractant protein-1, MIP-1α—macrophage inflammatory protein-1α, MIP-1β—macrophage inflammatory protein-1β, RANTES—regulated upon activation, normal T cell expressed and presumably secreted, IL-8—interleukin-8, MIG—monokine induced by γ interferon, IP-10—interferon-γ inducible protein 10kDa, I-TAC—interferon–inducible T cell α chemoattractant, FKN—fractalkine, a—*p* < 0.05; Mann–Whitney *U*-test, *—Could not be properly evaluated.

**Table 4:** Stratification of studies per year of publication based on design, allocation, and strain with the main results that highlight inflammation in rats and mice.

	*Rats*				
Number of Experimental Models and Allocation	Strain	Inflammatory Biomarker	*p*-Value	Method of Assessment	Year of Publication and Reference
n = 21 n = 7 bilateral sham vasectomy/unilateral sham inoculation n = 7 bilateral sham vasectomy/unilateral inoculation of *Escherichia coli* n = 7 bilateral vasectomy/unilateral inoculation of *Escherichia coli*	Sprague-Dawley	Controls IL-1α (pg/µL) 53.8–60.0 pg/µL IL-1β (pg/µL) 78.8–83.6 pg/µL IL-4 (pg/µL) 232.4–240.0 pg/µL	< 0.05	bead-based multiplexed immunoassay	2011 [156]
	***Mice***				
n = 56 n = 8 untreated group n = 8 shamVx+PBS group n = 10 Vx+PBS group n = 8 shamVx+TGC group n = 22 Vx+TGC group	A/J	testis shamVx+TGC group IFNγ IL-10 epididymis Vx+PBS group IFNγ IL-6 IL-10 Vx+TGC group IL-6 IL-10 all are relative to β-actin	testis < 0.05 epididymis Vx+PBS group < 0.05 Vx+TGC group < 0.01	mRNA by real-time RT-PCR	2008 [155]
n = - n = - INT n = - SVX n = - VAS	BALB/c (H-2 ^d^)	capacitated sperm a2v Lif IL-1β TNF a2NTD injected horn Lif IL-1β TNF Ccl2 INT day 4 a2v Ccl2 Lif Hoxa 10 - all are relative to Gapdh	capacited sperm < 0.01 a2NTD injected horn < 0.01 INT day 4 a2v, Ccl2 < 0.01 Lif, Hoxa 10 < 0.05	multiplex real-time PCR	2012 [170]
n = 20 n = 10 sham group n = 10 vasectomized	BALB/c	Galectin-3	-	-	2013 [168]

RT-PCR—real-time polymerase chain reaction, Ccl2—C-C motif chemokine ligand 2, Lif—leukemia inhibitory factor, a2V—a2 vacuolar-ATPase, HOXA 10—homeobox protein hox-A10

**Table 5:** Leading human semen microbiota studies based on the allocation of patients and platform used for sequencing with the microbial region and oscillations within communities observed in human individuals.

Total Number of Patients and Allocation	Hypervariable Region	Sequencer	Microbial Ratio	Year of Publication	Reference
n = 37 n = 25 idiopathic infertility men n = 12 healthy men	V3-V4	MiSeq—Illumina	*Collinsella* ↓↑ *Staphylococcus* ↓ *Lachnospiraceae* ↑ *Coprococcus* ↑ *Anaerococcus* ↓↑	2021	[53]
n = 58 n = 18 post-vasectomy men n = 22 fertile non-vasectomized men n = 18 post-vasectomy men	V12	MiSeq—Illumina	*Sphingomonas* ↓ *Brevundimonas* ↓ *Paracoccus* ↓ *Corynebacterium* ↑	2023	[187]

↓—decreased, ↑—increased, ↑↓—fluctuations.

## Data Availability

The datasets used and analyzed during the current study are available from the corresponding author on reasonable request.
